# Adjunctive imprint cytology of core needle biopsy specimens improved diagnostic accuracy for breast cancer

**DOI:** 10.1186/2193-1801-2-372

**Published:** 2013-08-06

**Authors:** Shinichiro Kashiwagi, Naoyoshi Onoda, Yuka Asano, Satoru Noda, Hidemi Kawajiri, Tsutomu Takashima, Masahiko Ohsawa, Seiichi Kitagawa, Kosei Hirakawa

**Affiliations:** Department of Surgical Oncology, Osaka City University Graduate School of Medicine, 1-4-3 Asahi-machi, Abeno-ku, Osaka, 545-8585 Japan; Department of Diagnostic Pathology, Osaka City University Graduate School of Medicine, 1-4-3 Asahi-machi, Abeno-ku, Osaka, 545-8585 Japan; Department of Physiology, Osaka City University Graduate School of Medicine, 1-4-3 Asahi-machi, Abeno-ku, Osaka, 545-8585 Japan

**Keywords:** Adjunctive imprint cytology, Biopsy, Breast cancer, Diagnosis, False negative

## Abstract

**Objective:**

Recently, therapies targeting the biological characteristics of individual cancers according to markers indicating underlying molecular biological mechanisms have become available. Core needle biopsy (CNB) is widely used, not only to diagnose, but also to determine therapeutic strategies, in patients with breast cancer. Although the diagnostic accuracy of CNB is acceptably high, false-negative results have occasionally been encountered.

**Methods:**

The results of adjunctive imprint cytology (AIC) coinciding with CNB in 2,820 patients suspected to have breast cancer were retrospectively reviewed. The feasibility and clinical usefulness of AIC-assisted diagnosis were analyzed.

**Results:**

Fourteen-hundred and sixty-four cases were diagnosed as not malignant using CNB alone. Forty-seven of 1464 cases were suspected to be malignant on a cytological review of AIC, and 42 were confirmed to be breast cancer on additional biopsies. The combination of CNB and AIC achieved a sensitivity of 100% (1398/1398) and a specificity of 99.6% (1417/1422). Small lesions and large noninvasive- or scirrhous-type carcinomas were the common features of the CNB-negative/AIC-positive cases.

**Conclusions:**

Adjunctive imprint cytodiagnosis is a simple and easy procedure that assists the pathological diagnosis of breast cancer using CNB and therefore serves as a possible novel standard application.

## Introduction

Recently, molecular biological mechanisms involved in the growth and metastasis of breast cancer have been intimately elucidated. Therapies targeting the biological characteristics of individual cancers according to markers indicating underlying molecular biological mechanisms have become available. For instance, hormone, chemo and HER2/neu molecular-targeting therapies are widely used based on the expression states of estrogen receptor (ER), progesterone receptor (PR), Ki67 and the HER2/neu expression status in cancer cells (Sorlie et al. 2003; Bauer et al. 2007; Rastogi et al. 2008; Perez et al. 2010; Abd El-Rehim et al. 2005; Mattie et al. 2006). Moreover, the expressions of these molecules are also known to have roles as prognostic indicators (Rakha et al. 2006; Sørlie et al. 2006; Rakha et al. 2007). Hence, molecules used as therapeutic targets, as well as prognostic predictors, can be determined prior to the initiation of tailor-made therapies. In order to obtain such molecular information and determine an accurate pathological diagnosis, acquiring sufficient amounts of tissue samples from individual tumors is required. Although open biopsy and Mammotome® (MMT) are the best procedures to acquire large amounts of tissue samples (Brem et al. 2001; Verkooijen 2002; Hoorntje et al. 2003), it is not practical to apply these invasive and expensive procedures to every tumor suspected of being breast cancer. The type of subjects considered for these procedures should therefore be limited.

For these purposes, CNB (core needle biopsy) is universally performed to diagnose breast cancer. We have been employing CNB as a routine diagnostic procedure in our institute since 2007. For the experienced hand, performing the CNB procedure is very easy, minimally invasive, safe and inexpensive when compared with open biopsy or MMT (Brem et al. 2001). Previous reports of CNB have shown a sensitivity of as high as 86–90% and a specificity of 89–96% (Westenend et al. 2001; Hatada et al. 2000; Ballo & Sneige 1996). Furthermore, the precise characteristics of cancer cells can be investigated from the tissue samples obtained with CNB (Varga et al. 2005; Taucher et al. 2003; Cahill et al. 2006).

However, at the same time, false-negative results have occasionally been encountered in the diagnosis of breast cancer using CNB (Varga et al. 2005; Taucher et al. 2003). The obtained tissue samples are small, and it is sometimes difficult to demonstrate a suitable cut surface on a slide for pathological investigation. Therefore, most diagnostic errors from CNB result from failures to investigate whole specimens due to improperly obtained samples or inadequately presented histological specimens. To improve the diagnostic accuracy of CNB, adjunctive imprint cytodiagnosis (AIC) has been used in our institute. We experienced a number of cases in which significant benefits were gained using this procedure.

We herein retrospectively reviewed and evaluated the usefulness and availability of AIC-assisted CNB for making accurate diagnoses of breast cancer.

## Materials and methods

### Patient background

The subjects included 2,720 consecutive patients suspected to have breast cancer who underwent CNB and imprint cytodiagnosis at our department between May 2007 and March 2013. Suspicion of the disease was determined on either physical examination, CT scan, magnetic resonance imaging or US. Every lesion was clearly demonstrated and fully investigated on US before any biopsies were performed. Only patients from whom written informed consent was adequately obtained by explaining the procedures and possible outcomes were included. We performed biopsies in cases of category 4–5 BI-RADS classification (Ball et al. 2002; Orel et al. 1999). Several patients with tumors classified as category 3 were also included according to the recommendations of supervising doctors. One-hundred and seventy two patients were excluded from undergoing CNB and directly sent to undergo vacuum-assisted biopsies such as MMT or the self-contained vacuum-assisted biopsy system (Vacora®). These patients were suspected to have ductal carcinoma in situ with predominant US findings of ductal dilatation without clear tumor formation. No complications were observed in association with the procedures. No patient diagnosed with benign disease developed malignant disease during the follow-up period of two to 45 months.

### Method of CNB and adjunctive imprint cytology (AIC)

**Figure 1:**
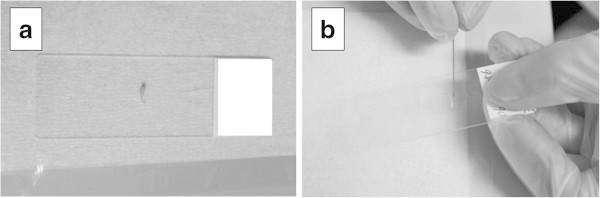
**Core needle biopsy and adjunctive imprint cytology methods.** In principle, two biopsies were sampled from the each lesion **(a)**. Immediately after the tissue sample itself and the needle were touched they were rolled over on a slide glass and fixed with 95% ethanol **(b)**.

In each patient, local infiltration anesthesia was administered with 0.5% epinephrine-containing lidocaine hydrochloride for the skin and puncture pathways and around the lesions. Then, 1.5 mm of skin was excised for biopsy using an ultrasound-guided 16 G biopsy needle. In principle, two biopsies were sampled from each lesion (Figure 1a). In each case, immediately after the tissue sample was taken out from the needle, the tissue sample and the needle were touched and rolled over on a slide glass and fixed with 95% ethanol (Figure 1b). A cytological diagnosis was made based on the investigation of Papanicolaou stained specimens by approved cytologists and cytopathologists. The specimens were classified into five categories according to the general rules of clinical and pathological recording of breast cancer (Wang & Ducatman 1998): “normal or benign”, “indeterminate”, “suspicious for malignancy” and “malignant”. The tissue samples were fixed in 10% buffered formalin. Several serial HE-stained slides were obtained from formalin-fixed and paraffin-embedded tissue samples for pathological examination by more than two pathologists. For the tissue samples diagnosed as cancerous, immunohistochemical staining against ER, PR, Ki67 and HER2/neu was conducted in addition to the routine pathological diagnosis.

## Results

**Figure 2:**
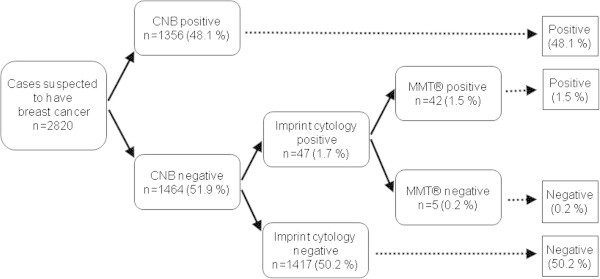
**Schema of our diagnostic procedure was shown.** A CNB diagnosis demonstrated that, of all the 2,820 cases, 1464 (51.9%) were benign and 1356 (48.1%) were malignant. Forty-seven cases (1.7%) were diagnosed as benign by CNB, but suspected of being malignant by AIC. Finally, 42 of the 47 patients (89.4%) were revealed to have breast cancer.

A review of the diagnoses made with CNB demonstrated that, of all the 2,820 cases, 1,464 (51.9%) were benign and 1356 (48.1%) were malignant. CNB alone showed a sensitivity of 96.6% (1351/1398), a specificity of 99.6% (1417/1422) and a false-negative rate of 3.4% (47/1398). Of the malignant cases, 47 (1.7%) were diagnosed as benign on CNB and suspected of being malignant on AIC. Ultimately, 42 of these 47 patients (89.4%) were revealed to have breast cancer (Figure 2).

Table 1 shows the clinico-pathological backgrounds of the 42 cases diagnosed to have breast cancer on AIC. All of the patients were female. Their ages ranged from 30 to 82 (mean: 61.0) years. Thirteen patients were pre-menopausal and twenty-nine patients were post-menopausal. The tumor diameters measured between 0.4 and 8.0 (mean: 1.9) cm. Nine cases involved tumors measuring less than 1 cm in diameter and 26 (61.9%) cases involved tumors measuring less than 2 cm in diameter. In sixteen cases (38.1%), no tumors were found within the tissue samples obtained with CNB. Ten cases (23.8%) were diagnosed as having benign hyperplastic lesions such as adenosis or hyperplasia. And two cases (4.8%) were necrotic tissue. Atypical cell clusters were found in the CNB specimens of the fourteen remaining cases (33.3%). Twenty tumors were diagnosed as “suspicious for malignancy” and twenty-two tumors were diagnosed as “malignant” according to the AIC specimens. Diagnoses of breast cancer were obtained with MMT biopsies before surgery, and 33 invasive ductal carcinomas (78.6%) and nine non-invasive ductal carcinoma in situ (21.4%) were identified on the final pathological investigations of the surgical specimens. ER and PR were positively identified in 26 (61.9%) and 21 (50.0%) cases, respectively. HER2/neu was found to be overexpressed in 12 cases (28.6%).

**Table 1:** **Clinico-pathological characteristics of the patients and breast cancers identified by adjunctive imprint cytology of the core-needle biopsied specimens**

Age	Menopausal	CNB Dx.	AIC Dx.	Final Dx.	Size (cm)
46	After	No tumor	SM	IDC*	0.4
66	Post	No tumor	SM	DCIS**	0.5
76	Post	No tumor	SM	IDC	0.6
73	Post	Intraductal papilloma	M	DCIS	0.7
67	Post	Intraductal papilloma	M	IDC	0.8
70	Post	Atypical cell cluster	SM	IDC	1.0
56	Post	Atypical cell cluster	M	IDC	1.0
78	Post	No tumor	M	IDC	1.0
52	Post	Fibrocystic condition	SM	DCIS	1.0
46	After	Chronic mastitis	SM	IDC	1.2
41	After	No tumor	SM	IDC	1.2
68	Post	Apocrine metaplasia	M	DCIS	1.2
71	Post	Necrotic tissue	SM	IDC	1.2
67	Post	Atypical cell cluster	M	IDC	1.3
77	Post	Ductal hyperplasia	M	DCIS	1.5
51	After	No tumor	SM	IDC	1.5
33	After	Adenosis	M	IDC	1.6
59	Post	No tumor	SM	IDC	1.6
51	Post	Atypical cell cluster	SM	IDC	1.6
43	After	No tumor	SM	DCIS	1.7
75	Post	Atypical cell cluster	M	IDC	1.8
30	After	Atypical cell cluster	M	IDC	1.9
58	Post	Atypical cell cluster	M	IDC	1.9
75	Post	No tumor	SM	IDC	1.9
74	Post	Fibrocystic condition	SM	IDC	2.0
53	Post	Atypical cell cluster	SM	IDC	2.0
66	Post	Atypical cell cluster	SM	IDC	2.1
64	Post	Atypical cell cluster	M	IDC	2.2
43	After	Adenosis	M	IDC	2.4
42	After	No tumor	SM	DCIS	2.4
78	Post	Phyllodes suspicious	SM	IDC	2.4
82	Post	No tumor	M	IDC	2.4
72	Post	Atypical cell cluster	M	IDC	2.6
62	Post	No tumor	M	IDC	2.9
58	Post	No tumor	M	IDC	3.0
45	After	Atypical cell cluster	M	IDC	3.0
54	Post	No tumor	SM	IDC	3.5
52	Post	Atypical cell cluster	M	IDC	3.5
47	After	No tumor	SM	IDC	3.7
45	After	Atypical cell cluster	M	DCIS	4.0
52	After	No tumor	M	DCIS	5.0
77	Post	Necrotic tissue	M	IDC	8.0

*CNB* core-needle biopsy, *Dx.* diagnosis, *SM* suspicious for malignancy, *M* malignant, *AIC* adjunctive imprint cytology of the core-needle biopsied, *IDC: invasive ductal carcinoma, **DCIS: ductal carcinoma in situ.

In contrast, five tumors (10.6%) classified as “suspicious for malignancy” on AIC were ultimately revealed to have been diagnosed correctly in the initial pathological review of the CNB specimens. The final pathological diagnosis in five cases was confirmed using MMT biopsies to be benign disease (three cases of fibroadenoma and two cases of intra ductal papilloma). Therefore, of all the 2,820 cases, 1,422 (50.4%) were ultimately diagnosed as benign and 1,398 (49.6%) were ultimately diagnosed as malignant. Forty-two false-negative cases on pathological review of CNB and five false-positive cases on cytological review of AIC were found. The combination of CNB and AIC achieved a sensitivity of 100% (1398/1398) and a specificity of 99.6% (1417/1422) (Table 2).

**Table 2:** **The results of combination diagnosis with the core-needle biopsy and the s adjunctive imprint cytology**

CNB/AIC	Benign	Cancer	Total
Both negative	1417	0	1417
Either positive	5	1398	1403
Total	1422	1398	2820

*CNB* core-needle biopsy, *AIC* adjunctive imprint cytology of the core-needle biopsied specimen.

**Figure 3:**
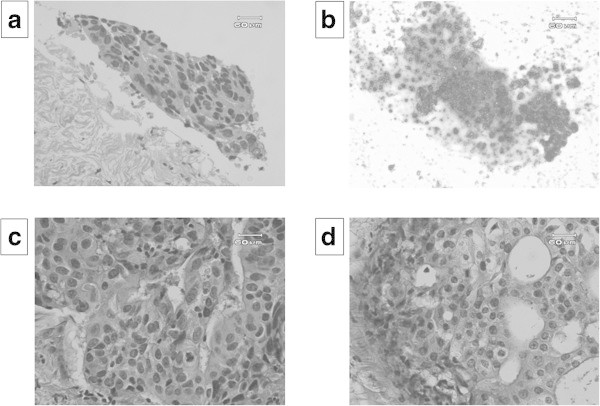
**A false negative case was demonstrated.** A small area of atypical cell cluster was found in the CNB specimen taken from a 58 years-old woman with right breast tumor of 1.9 cm in maximal diameter **(a)**. Adjunctive imprint cytology revealed cancerous cells with papillary clumping **(b)**. Invasive ductal carcinoma was clearly demonstrated in Mammotome® **(c)** and resected specimens **(d)**.

Figure 3 shows a representative case. A 58-year-old female was found to have a 1.9 cm tumor on her right breast on US. She underwent CNB on suspicion of having breast cancer. Only a small area of atypical cell clusters was demonstrated in the CNB sample, which was not considered an adequate quantity to diagnose the lesion as breast cancer. On the other hand, AIC classified the tumor as “malignant” based on the demonstration of cancerous cells with papillary clumping. A final diagnosis of invasive ductal carcinoma was made using MMT. This diagnosis was consistent with the pathological features of the surgically resected specimen.

## Discussion

A considerable number of cases have shown discrepancies between clinical images and pathology in the diagnosis of breast lesions. Nevertheless, in cases with strong clinical suspicion of malignancy, most lesions that cannot be proven to be pathologically malignant on biopsy are ultimately revealed to be benign. However, on some occasions, malignant disease can only be identified after repeated investigation, resulting in critical delays in initiating anti-cancer therapy. To avoid these problems, sufficient specimens should be presented for pathological review. Open biopsy or MMT are useful methods for obtaining adequate tissue samples to make accurate pathological diagnoses (Brem et al. 2001; Verkooijen 2002; Hoorntje et al. 2003). However, it is not practical to perform these invasive and expensive procedures in every patient, and the simple CNB technique is universally used as a routine method of tissue biopsy in diagnosing the majority of cases of breast cancer (Westenend et al. 2001; Hatada et al. 2000; Ballo & Sneige 1996). Therefore, maintaining the accuracy of pathological diagnosis with CNB at the highest level is important.

Previous reports describing the accuracy of pathological diagnosis with CNB have shown sensitivities of as high as 86–90% and specificities of 89–96% (Westenend et al. 2001; Hatada et al. 2000; Ballo & Sneige 1996). In this study, our experience using CNB alone showed a sensitivity of 96.6%, a specificity of 99.6% and a false-negative rate of 3.4%, which was in line with the results of previous reports. According to these observations, the diagnostic accuracy of breast cancer with CNB seems to have already reached a peak. Although this level of diagnostic accuracy is acceptable, it is true that occasional false-negative cases still occur.

One of the most compelling explanations for the occurrence of false-negative CNB results is the unintentional loss of the target lesion. Occasionally, only small cancerous tissues are included in CNB specimens, as shown in our case. Even with careful handling, lesions can separate from the specimens during the multitude of steps of tissue processing. However, AIC performed immediately after sampling using a simple imprinting method maintains the cancerous cells within the samples, which supplements the demerits of CNB.

Several tumor features are known to exert negative impacts on making an accurate CNB diagnosis of breast cancer. In many cases of non-invasive ductal carcinoma, the tumors could not be identified clearly on US (Evans 1992) or were found to display predominantly para-tumoral features such as ductal dilatation or irregularities (Li et al. 2010). All of these features can be evaluated before performing CNB, and alternate methods should be implemented such as MMT or Vacora® (Salem et al. 2009; Wang et al. 2009; Hauth et al. 2008), as stated in the text. On the other hand, several factors might disturb the process of acquiring appropriate tissue specimens for CNB, even when a clear view of the subject is achieved. Sometimes, needles slip on surfaces or cannot penetrate hard lesions (Ljung et al. 2001). The inability to prevent the patient from moving or to secure the puncture devise might result in the failure to obtain tumor-rich material (Ljung et al. 2001). However, even on these occasions, cancer cells can be identified on the surface of the tissue samples or the needle and can be demonstrated on AIC slides.

The stroke of the needles used in this study was 22 mm long. Therefore, non-tumorous tissues were often included when performing CNB of small lesions. Similar problems appeared when the tumors consisted of mixtures of scattered cancer cells and matrices, such as in cases of non-invasive carcinoma or sclerosing type (scattered cancer cells accompanying predominant fibrotic tissues) carcinoma. On these occasions, the cancer nests occupied only small parts of the specimens, and the targets could not be presented appropriately on the tissue slides for microscopic examination. For these lesions, AIC might therefore have been significantly effective.

The use of cytological examination has critical demerits for investigating the tissue structures of lesions, an important factor used to distinguish breast cancer from benign disease (Ogawa et al. 1998). The experience and individual particularity of the cytologist may increase the false-positive rate of cytological diagnosis, especially for organs, such as the mammary glands, that are highly dependent on hormonal circumstances (Ljung et al. 2001; Pisano et al. 2001; Giard & Hermans 1992). Therefore, problems remain in the diagnosis of breast cancer using FNA alone. At the same time, cytological diagnosis has superiorities in its ability to oversee whole specimens at a glance, its facility and its non-invasiveness. In several previous reports, the usefulness of AIC for diagnosing cancer involvement in the sentinel lymph nodes has been described (Motomura et al. 2000; Llatjós et al. 2002; Lee et al. 2002). A similar technique was used in the present study in order to supplement the weak points of pathological diagnosis using CNB.

The use of AIC in combination with CNB does not add any burdens to the patient’s body and can be performed with small expense. The procedure is not complicated and only requires rolling the tissue samples on the slide glass while confirming whether the obtained tissue specimens are sufficient. Moreover, we demonstrated significant clinical benefits of AIC in improving the pathological diagnosis of breast cancer.

In the present study, we described 42 cases of breast cancer identified using AIC in combination with CNB. No special features in the backgrounds of the patients were observed, indicating the universal applicability of this simple method. More than half of the tumors in the present study measured less than 2 cm in diameter, and all tumors measuring more than 2 cm in diameter showed either ductal carcinoma in situ or sclerosing type, histologically. As described earlier, it has been clearly demonstrated that AIC has a significant additional impact for diagnosing lesions with characteristics that indicate possible difficulties in making an accurate diagnosis with CNB alone. In this study, we experienced five false-positive cases with AIC. After confirming the final pathology using tissue obtained by MMT, both cases were revealed to have been diagnosed correctly on CNB. The diseases observed in these five cases (three cases of fibroadenoma and two cases of intra ductal papilloma) tend to be overly diagnosed using cytology (Giard & Hermans 1992). It may be possible to omit performing MMT with more intimate mutual consultations between surgeons and pathologists.

We herein retrospectively examined the usefulness and applicability of adjunctive imprint cytodiagnosis of core needle biopsied specimens in the diagnosis of breast cancer and found significant benefits for this technique in improving the ability to make an accurate diagnosis. To our knowledge, no previous reports have been published on these issues. Adjunctive imprint cytodiagnosis is a simple and easy procedure that assists the pathological diagnosis of breast cancer with CNB and therefore serves as a possible novel standard application.
